# Evaluation of a simplified pharmacovigilance tool for general practitioners: 5 years of insight

**DOI:** 10.1038/s41598-024-51753-w

**Published:** 2024-01-20

**Authors:** A. Trenque, A. Rabiaza, S. Fedrizzi, B. Chretien, M. Sassier, R. Morello, J. Alexandre, X. Humbert

**Affiliations:** 1 Department of Pharmacology, CHU de Caen-Normandie, 14000 Caen, France; 2 Department of General Medicine, Normandie Univ, UNICAEN, UFR Santé, 2, Rue des Rochambelles, 14000 Caen, France; 3 Biostatistics Unit, Clinical Research Department, CHU de Caen Normandie, 14000 Caen, France; 4 UNICAEN, INSERM U1086 ANTICIPE, Normandie Univ, 14000 Caen, France

**Keywords:** Epidemiology, Drug development

## Abstract

Spontaneous reporting of adverse drug reactions (ADRs) is the cornerstone of pharmacovigilance. However, major underreporting exists. The main objective of this study was to assess the use of a pharmacovigilance simplified reporting tool (PSRT) by general practitioners (GPs) and, secondarily, to describe the quality of ADR reports during this period. The PSRT was proposed on June 1st, 2015, for the 1290 GPs in the Western Normandy Region. The number and quality of ADRs reported monthly by GPs were prospectively collected from June 1st, 2015, to May 31st, 2020 (Period 2), and compared to those reported during a control period (June 1st, 2010, to May 31st, 2015, Period 1). During all the periods, 920 reports were made by 307 GPs (198 reports in Period 1 and 722 reports in Period 2), with 477 reports (51.8%) using the PSRT. During Period 2, the monthly number of reports was multiplied by 3.5 (p < 0.0001), and the number of GPs was 1.4 compared to that in Period 1 (p = 0.01). Our PSRT showed effectiveness in quantitative and qualitative terms. It must now go further and be integrated into GP software to facilitate ADR reporting nationwide.

## Introduction

Postmarketing drug safety surveillance was implemented to ensure the safety of drugs. Surveillance monitors a drug from marketing authorization throughout its time on the market. The French pharmacovigilance (PV) system is composed of a network of regional pharmacovigilance centres (CRPVs). They are located inside university hospitals and are coordinated by the *Agence nationale de sécurité du médicament* (ANSM)^1^.

In practice, postmarketing drug safety surveillance is primarily based on spontaneous reporting of adverse drug reactions (ADRs) to CRPVs. These reports are the cornerstone for PV actions and studies. Even if the reporting rate is low^2^, spontaneous reporting allows the emergence of safety signals^1^. In addition to its low cost and ease of use^1^, this approach can lead to improvements in the risk–benefit balance of drugs and can ultimately lead to commercialization^3^. Thus, CRPVs collect and analyse the reported ADRs. For each patient, the causal link between the incidence of the ADR and drug intake is evaluated. These reports describe an ADR suspected to be caused by one or more drugs. The value of these reports is even more relevant because they contain large amounts of clinical data^4,5^. Without these data, the link between suspected drug(s) and ADRs is much more difficult to determine. Safety drug agencies often point out the utility of a high-quality management system for these reports^6,7^.

In France, prescribers (physicians, dentists and midwives) and pharmacists, in theory, have to report all ADRs or suspected ADRs when aware of them. Other health professionals can also report ADRs when they are aware of them^8^. Moreover, since 2011, patients have reported ADRs as well. In this context, general practitioners (GPs) are among the major actors in primary care. However, they made only 7% of the ADR reports in France in 2014^1^, and according to a French study, GPs report only 1 out of every 24,433 ADRs to their local CRPV^9^. On the other hand, GPs prescribe three quarters of the drugs delivered in France^10^. Faced with this underreporting (mainly due to lack of time^11^), the Department of General Practice (DGP) of the University of Caen-Normandy and the CRPV of Caen-Normandy University Hospital have developed a PV simplified reporting tool (PSRT) dedicated to ADR reports for GPs and hosted by the Normandy Regional Union of Liberal Physicians (URML Normandie) for the subdivision of Caen (western Normandy). We collected the first insights after the first years of use (1 and 3 years of follow-up). This new tool has permitted us to increase the monthly number of reports coming from GPs (by 4.8 and 3.0, respectively) and the number of reporting GPs. The quality of reporting was also unchanged during the same follow-up period. After this first encouraging track record, longer-term insight is necessary^12,13^.

In this way, the aim of this study was to assess the use of this PSRT during 5 years by GPs in terms of the number of ADRs reported compared to a 5-year control period and, secondarily, to describe the quality of ADR reports during this period.

## Methods

The PV reports can be sent to the CRPV by any means. The aim of our PSRT was to make the PV report during the face-to-face consultation by the GP with the patient who presented the ADR. A minimum of pertinent information collected by GPs was needed to ensure that the quality of the report would not be degraded by the PSRT.

### Characteristics of the simplified report

**Figure 1:**
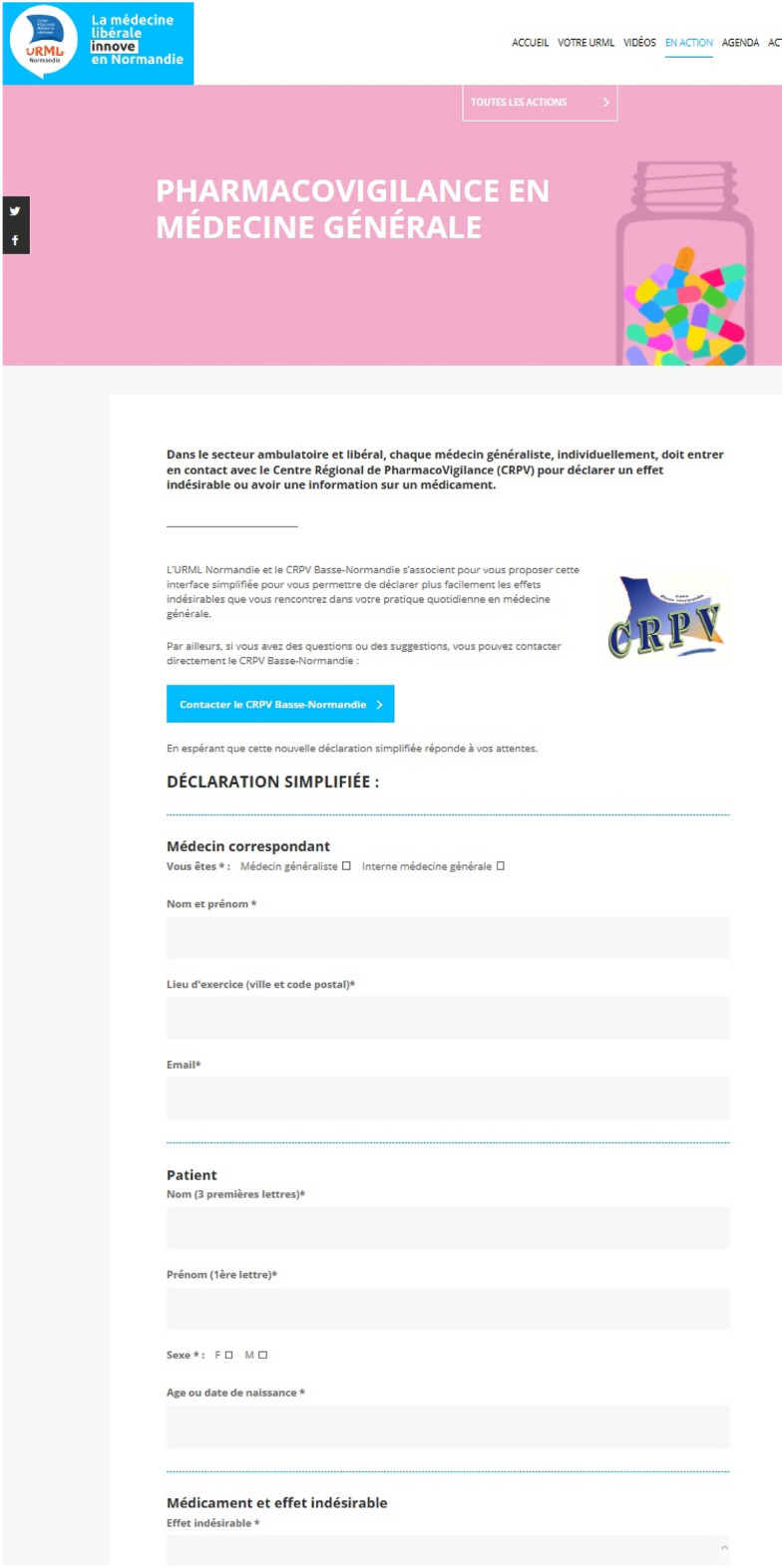
The simplified online tool for pharmacovigilance is hosted on the website of the URML Normandie (available on: https://www.urml-normandie.org/en-action/pharmacovigilance-en-medecine-generale/).

After a concertation at the CRPV of Caen (4 pharmacologists) with the DGP of Caen (2 GPs), the key information to collect from the PSRT was defined, in line with the French imputation of the unexpected or toxic effects of drugs method^14^ and recommendations of the European Medicine Agency (EMA)^15^, as follows (Fig. 1):

- GP information: name—practice address—email address;
- Patient information: surname (3 first letters)—first name (first letter)—sex—age;
- The drugs taken and the adverse events reported; and
- Pharmacy who made the delivery to retrieve all the data about regular past drug deliveries: name—address.
- Attachments (photos, consultation reports, etc.) could be added.

Apart from the attachment files, all other fields were required to be able to validate and send the report. For example, pharmacists at the CRPV could contact GPs by email to improve precision (for example, through the evaluation of chronologic imputability).

This PSRT was promoted by the URML Normandie on its mailing list. Its mailing list is exhaustive and contains all GPs who work in western Normandy. An email was sent to all GPs on June 1st, 2015, and iterative reminders were sent (June 8th, 2016; October 9th, 2017; May 15th, 2018; and October 28th, 2019).

### Data selection

In this study, reports made by GPs between June 1st, 2010, and May 31st, 2020, were extracted from the local database of the CRPV of Caen. We performed a descriptive analysis of the number and general characteristics of the ADRs reported during this period by GPs to the CRPV of Caen before (June 1st, 2010, to May 31st, 2015, Period 1) and after (June 1st, 2015, to May 31st, 2020, Period 2) the implementation of the PSRT (June 1st, 2015). ADR reports correspond to notifications of ADRs. The types of declarations are also listed.

For each report, the collected data included patient age and sex, type of ADR classified according to the Medical Dictionary for Regulatory Activities (MedDRA)^16,17^, suspected drug classified according to the Anatomical Therapeutic Chemical (ATC) classification^18^ and severity of the ADR. An ADR was considered “serious” if the result was one of the following situations: death, life-threatening conditions, hospital stay, longer hospital stay, incapacity, persistent invalidity, major anomaly or congenital malformation^4^. A notification is the transmission of an ADR of a suspected drug or product to a PV structure or to the ANSM^19^. It is important to note that adverse events are reported to CRPVs and not ADRs or suspected ADRs. Indeed, an adverse event is classified as an ADR once imputability (causality assessment) is performed to confirm a causal link between the adverse event and the suspected drug.

We excluded reports related to specific ADR reporting channels (cardio-oncology program PICARO and the addictovigilance program of Caen-Normandy University Hospital, France) and details queries (DQs) without ADRs.

### Measurement of the influence of the PSRT on pharmacovigilance

To evaluate the influence of this new PSRT, we used the rate of ADRs reported by GPs, which was related to the totality of ADRs reported to the CRPV in Caen before (Period 1) and after (Period 2) the introduction of the PSRT. We also compared the monthly average number of reports by GPs and of GPs making reports between these two periods. We also compared the number of reports not made by the PSRT between these two periods. Moreover, we compared the reporting GPs and patient characteristics and parameters such as ADR severity according to the mode of reporting chosen by the GP during all the periods. Finally, we studied the rate of ADRs reported to the CRPV in Caen compared to that reported to the nationwide CRPV in terms of GP and all reports during the two study periods.

### Evaluation of the quality of the pharmacovigilance reports

According to the recommendations of the EMA concerning good PV practices^15^, an ADR report is considered valid if it includes an identifiable reporter, an identifiable patient (initial, identification number, date of birth, age, sex), at least one suspected ADR and one suspected drug. A lack of one of these elements means incomplete observation. In addition to these mandatory elements, a well-documented report must include the medical and disease histories, the other drugs taken concomitantly, the results of complementary explorations in relation to the ADR, therapeutic management, disease evolution and response after stopping and/or reintroducing the suspected drug.

In our study, based on these good PV practices^15^ and the literature^1^, the data were classified as mandatory or optional. The mandatory criteria included (1) patient identification (identification number), (2) age, (3) sex, (4) ADRs and (5) suspected drug(s).

The optional criteria included (1) disease history, (2) concomitant treatments, (3) symptom evolution and (4) complementary exploration or nondrug diagnosis results.

The reports were then classified according to the presence or absence of the mandatory and optional items:

- “well-documented” reports: if all 5 mandatory items and all 4 optional items were completed;
- “slightly documented” reports: if all 5 mandatory items were completed and one optional item was missing;
- “poorly documented” report: all other situations.

We studied the quality of the reports, regardless of type, during the two follow-up periods. Moreover, to evaluate the quality of the new PSRT reports, we compared them to reports made in a standard way during the entire follow-up period, with percentage comparisons or means.

### Legal aspects

The study protocol received favourable notice from the *Commission nationale de l'informatique et des libertés*, the French data protection authority (number 1815708v0), on December 3rd, 2014, to confirm that all methods were carried out in accordance with relevant guidelines and regulations. Informed consent was obtained from all subjects and/or their legal guardian(s).

### Statistical analysis

Percentage comparisons were performed with the chi-square test, and mean comparisons were performed with Student’s t test on NCSS software (NCSS 12 Statistical Software (2018), NCSS, LLC, Kaysville, Utah, USA, ncss.com/software/ncss). In addition, we used Pearson correlation analysis to study hypothetical differences in PV reporting between the two periods considered and within each period. A result was considered statistically significant if the p value was less than 0.05.

## Results

### Reporters’ general characteristics (Table 1 and Fig. 2)

**Table 1:** General characteristics of the reports in general practice from the CRPV of Caen according to the study period.

	Before the PSRT—Period 1 (n = 198)	After the PSRT—Period 2 (n = 722)	Total (n = 920)	
Number of reporters [n (%)]	144 (46.9%)	204 (66.4%)	307	p = 0.01
GP sex: women [n (%)]	57 (39.6%)	91 (44.6%)	139 (45.3%)	p = 0.62
Patient age [years (min–max)]	51.8 (3 weeks–96 years)	57.1 (1 month–99 years)	55.1 (3 weeks–99 years)	p = 0.02
Patient sex: women [n (%)]	118 (59.6%)	417 (57.8%)	535 (58.2%)	p = 0.70
Severity^a^ [n (%)]	82 (41.4%)	160 (22.2%)	242 (26.3%)	p < 10^–11^
Way of reporting				p < 10^–11^
PSRT	0 (0.0%)	477 (66.1%)	477 (51.8%)	
Email	30 (15.1%)	110 (15.2%)	140 (15.2%)	
Postal mail	88 (44.4%)	50 (6.9%)	138 (15.0%)	
Phone	64 (32.3%)	15 (2.1%)	79 (8.6%)	
Health ministry website	0 (0.0%)	35 (4.8%)	35 (3.8%)	
Fax	10 (5.1%)	24 (3.3%)	34 (3.8%)	
Visit	5 (2.6%)	11 (1.6%)	16 (1.7%)	
Unknown	1 (0.5%)	0 (0.0%)	1 (0.1%)	

GPs: general practitioners; PSRT: pharmacovigilance simplified report tool; n: number.

^a^An adverse drug reaction was considered “serious” if the result was one of these situations: death, life-threatening conditions, a hospital stay, a longer hospital stay, incapacity, persistent invalidity or a major anomaly or congenital malformation^4^.

**Figure 2:**
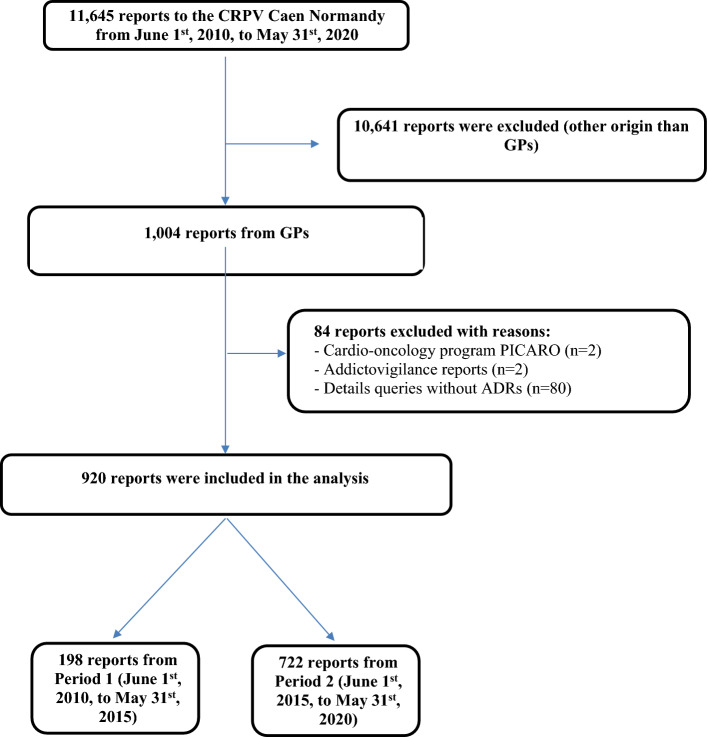
Flow chart of the study.

During the entire study period (Periods 1 and 2), 11,645 reports were registered in the CRPV of Caen, including 1004 from GPs (8.6% of the reports). We excluded 84 reports: 80 DQs without ADRs (22 during Period 1 and 58 during Period 2), 2 due to the cardio-oncology program PICARO (Period 2) and 2 concerning another regulatory vigilance system (addictovigilance) (Period 2) (Fig. 2). Consequently, 920 reports were analysed. During Period 1, there were 198 reports, and during Period 2, there were 722 reports after the application of the exclusion criteria.

During the entire study period (Periods 1 and 2), reports were essentially produced by the PSRT (51.8%, usable only during Period 2), followed by email (15.2%), postal mail (15.0%) and phone (8.6%). During the entire study period (Periods 1 and 2), 307 GPs (23.8% of 1290 GPs who worked in Western Normandy) published at least one report; 144 GPs participated during Period 1 and 204 during Period 2, and 41 GPs participated during both periods. The reporting GPs were mostly men (54.7%), with a sex ratio of 1.21 (Table 1).

**Figure 3:**
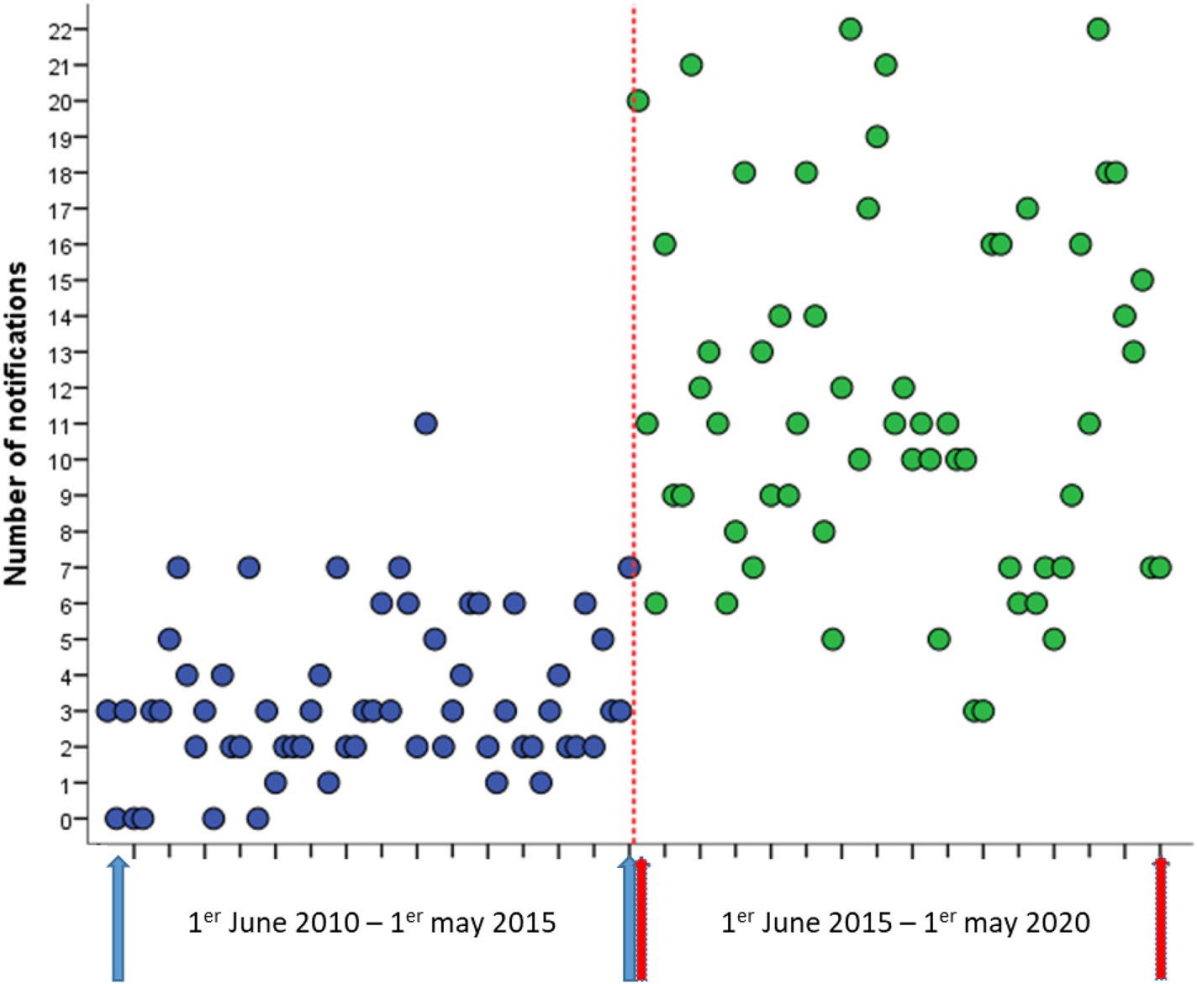
Pearson correlation analysis between the two periods considered.

During Period 1, the number of reports for one year was rather stable (35 in 2010–2011, 27 in 2011–2012, 49 in 2012–2013, 50 in 2013–2014, and 37 in 2014–2015), as in Period 2 (131 in 2015–2016, 132 in 2016–2017, 151 in 2017–2018, 156 in 2018–2019 and 152 in 2019–2020). During the entire study period (Periods 1 and 2), the monthly average number of reports was 8.3 during the entire follow-up. Pearson correlation coefficients were significantly different between the two periods considered (R = 0.697, p ≤ 0.001) but not within each period (Period 1: R = 0.217, p = 0.096; Period 2: R = − 0.061, p = 0.646) (Fig. 3).

### General characteristics of reports

On all the reports, 997 ADRs were reported, and in 1203 reports, one drug was suspected. The patients were mostly women (58.2%), with a mean age of 55.1 years (3 weeks–99 years). Almost a quarter of the reports were considered serious (26.3%).

The most common ADRs (classified by the MedDRA) were, in descending order, cutaneous affections (165, 16.6%), digestive affections (122, 12.2%), neurologic affections (111, 11.1%) and osteomuscular affections (92, 10.1%).

The most common suspected drugs were drugs for the cardiovascular system (259/1203, 21.5%), principally renin-angiotensin system antihypertensive drugs; nervous system drugs (226/1203, 18.8%), principally psychoanaleptics; and anti-infective drugs (206/1203, 17.1%), principally antibacterial agents.

During Period 2, patients were older than they were during Period 1 (57.1 versus 51.8; p = 0.02), and women composed the majority of patients (57.8%, p = 0.70). A significantly lower percentage of the reports were considered serious (22.2% versus 41.4%; p < 10^–11^).

### Influence of the PSRT

**Figure 4:**
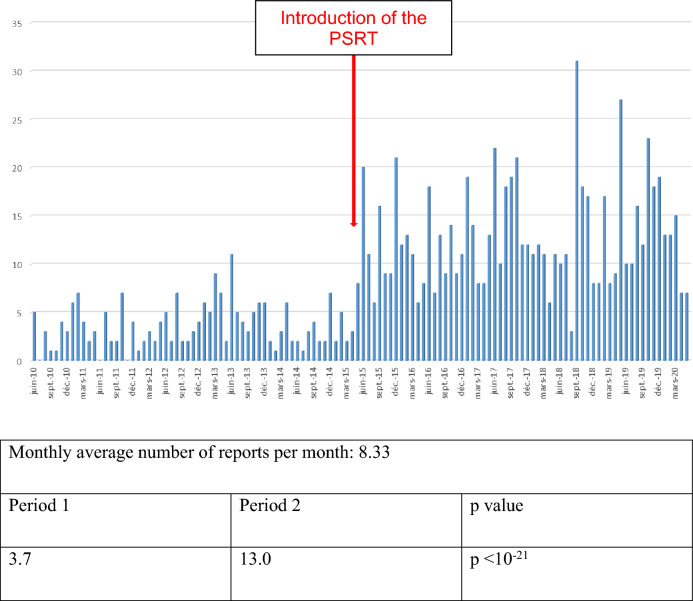
Monthly number of reports made before and after the introduction of the PSRT.

During Period 2, 3.5 additional reports (722 versus 198) and 1.4 additional reporting GPs, including 163 new reporters, were noted, particularly due to the PSRT. During Period 1, the average number of monthly reports was 3.7 versus 13.0 during Period 2 (p < 10^–21^) (Fig. 4). The reporting rate from GPs related to all reports concerning the CRPV in Caen was significantly greater (4.8% versus 9.5%, p < 0.0001). For ADR reports other than through the PSRT, the number increased to 23.7% (245 reports versus 198). This increase was larger than that for reports made by email (110 reports versus 30) (Table 1). During Period 2, regarding the general characteristics of the ADR reports made by the PSRT (Table 2), patients were older (57.4 years old versus 54.5 years old, p = 0.042), with fewer serious cases (16.6% versus 36.8%, p < 0.0001). Cutaneous ADRs were the most common. The most commonly suspected drugs were those for the cardiovascular system, followed by nervous system drugs and anti-infective drugs.

**Table 2:** General characteristics of the reports according to the way of reporting used in general practice.

	PSRT (n = 477)	Others (n = 443)	Total (n = 920)	p value
Age (years)	57.4 (1 month–98 years)	54.5 (3 weeks–99 years)	55.1 (3 weeks–99 years)	p = 0.042
Female sex [n (%)]	289 (60.6%)	293 (66.1%)	582 (63.3%)	p = 0.16
Severity^a^ [n (%)]	79 (16.6%)	163 (36.8%)	242 (26.3%)	p < 0.0001

PSRT: pharmacovigilance simplified report tool; n: number.

^a^An adverse drug reaction was considered “serious” if the result was one of these situations: death, life-threatening conditions, a hospital stay, a longer hospital stay, incapacity, persistent invalidity or a major anomaly or congenital malformation^4^.

### Evaluation of the quality of reports (Table 3)

**Table 3:** Quality of the reports made according to the period and the type of report.

	Well-documented	Slightly documented	Poorly documented	p value
Total [n (%)]	301 (32.7%)	301 (32.7%)	318 (34.6%)	
Considered period				p = 0.71
Period 1 n = 198	68 (34.3%)	60 (30.3%)	70 (35.4%)	
Period 2 n = 722	233 (32.3%)	241 (33.4%)	248 (34.3%)	
Type of report				p = 0.51
PSRT	152 (31.9%)	164 (34.5%)	160 (33.6%)	
Other reports	149 (33.6%)	137 (30.9%)	158(35.6%)	

PSRT: pharmacovigilance simplified report tool; n: number.

One-third of the reports were well documented according to our quality criteria. There was no significant difference in quality between the two periods (p = 0.71) or the type of declaration (PSRT/others) (p = 0.51) (Table 3).

### Number of reports in France in the same period (Table 4)

**Table 4:** Comparison of number of reports in France and at the CRPV of Caen.

	France	Caen	Total
Period 1	11,443	198	11,641
Period 2	15,146	722	15,868

Although the number of GP reports increased between the two periods in France (11,641 versus 15,868), the percentage of GPs decreased (6.3% versus 5.8%). Conversely, in the CRPV of Caen, the ratio of GP reports increased during Period 2 (4.8% versus 9.5%, p < 0.0001), as did the number of GP reports (198 versus 722).

## Discussion

After 5 years of the PSRT, we noted an increase of 3.5 in the number of reports and 1.4 in the number of different GPs reporting ADRs to the PV system, partly due to this new tool. This fact indicates that the PSRT maintained its utility during long-term periods both qualitatively and qualitatively. At the same time, in the CRPV of Caen, the number of GP reports and the ratio of reporting GPs increased as soon as the number of GP reports in France increased, but not the ratio of reporting GPs.

Our results are in line with those on the establishment of other systems, facilitating ADR reporting. Abadie et al.^20^ performed a study 18 months after the publication of an electronic reporting system in the CRPV of Toulouse in the Midi-Pyrénées region, France. They observed a 45% increase in the number of reports from private health professionals. Likewise, Biagi et al.^21^ showed a 200% increase in the number of ADR reports by Italian GPs after the creation of a monthly electronic newsletter about drug safety. However, their cohort included a low number of GPs (168/737, 22.8%). Finally, Durieu et al.^22^ reported twice the number of ADR reports at the CRPV of Toulouse, France, between 2013 and 2014 after the establishment of regular clinical research assistants visiting GPs in the Midi-Pyrénées region, France. In another study, the same team showed that these visits associated with a dedicated electronic system can increase the number of questions asked by GPs to the CRPV and improve patient care^23^.

In contrast, Johansson et al.^24^ did not observe any increase in the number of reports made by 151 primary health care units (GPs and nurses) in Region Västra Götaland, Sweden, after the publication of regular newsletters about ADRs.

In our study, one-third of the reports were well documented. There were fewer reports made using the PSRT, but the difference was not statistically significant. Durrieu et al.^25^ reported that, analysing 600 reports from GPs over 3 years, only one in eight were well documented.

Moreover, the introduction of this PSRT allowed an increase in the number of reports made by GPs without compromising their quality. Herdeiro et al.^26^ showed that attitudes and beliefs that generate underreporting are indulgence (believing that severe ADRs are well documented as soon as drugs are marketed), insecurity (believing that it is almost impossible to determine if a drug is responsible for a particular ADR), mistrust (believing that an ADR is reported only if we are sure that it is linked to the use of a particular drug), indifference (believing that one medical doctor’s case cannot contribute to medical knowledge) and ignorance (believing that only serious or unexpected ADRs have to be reported). In an Iranian study, Peymani et al.^27^ reported that PV underreporting by GPs was due to a lack of information on the reporting modality. They noted that only a quarter of the GPs had good knowledge about this topic when they sent a test about knowledge and procedures related to PV to 350 GPs. In this study, 91.3% of the GPs thought that ADR reports play a crucial role in decreasing the ADR incidence and improving patient safety. However, 68.5% of them said they did not know the function of the national Iranian PV centre. Additionally, in a study among 168 Italian GPs, Biagi et al.^21^ showed that 94% of GPs who had answered a form about PV judged that ADR reporting is an important part of their professional obligations, but 60% of them did not know that they could report even without a certitude of a causal relation between the drug and the ADR. Only 6.5% of the respondents provided a report within six months after receiving the form.

Other ways to improve ADR reporting in general practice, especially through the implementation of a practical format for GPs, have been evaluated^28,29^. Indeed, Gerritsen et al.^28^ compared the efficacy of an online method for training regarding PV focused on competencies, practice and a traditional method based on lectures in a Dutch GP professional course. This study showed a significant increase in the number and quality of PV reports by GPs who had followed the practical course. Additionally, a satisfaction study among GPs after the establishment of a network to help make PV reports showed a global satisfactory score of 9 out of 10^30^. For 91% of the respondent GPs, they considered that they had increased their number of reports since their participation in the network. Eighty-two percent of the participants thought that they would report ADRs more often in the future. This establishment allowed a time gain for 90% of the GPs and facilitated communication with the CRPV for 85%. This had a positive impact on their relationship with their patient due to an improvement in their vigilance about ADRs. Moreover, medical student sensitization is also likely relevant. Finally, a Dutch cohort study on postmarketing authorization for sumatriptan showed that patients reported more ADRs than did their GPs. Thus, a postmarketing authorization study that considers only ADRs reported by GPs can underestimate the incidence of ADRs, especially nonserious ADRs. It may be interesting to also send a form to patients through their GPs^29^.

This study showed that the most common drugs were related to the cardiovascular system, followed by those related to the nervous system and those related to preventing infection. These three drug classes were the most frequently encountered^9,20,22,23^. However, the most common antibiotics were antibiotics in a study by Leporini et al.^31^ and nervous system drugs in the studies by Abadie et al.^20^ and Moride et al.^9^. Additionally, in the literature, principal studies have shown that the most frequent ADRs are cutaneous affections, followed by digestive and neurologic affections^9,20,31^. These data are in line with our results.

The limitations of our study include the size of the sample and the low participation of the GPs. A total of 144 GPs provided information only during Period 1, while 41 did so during both periods. Retirement cannot explain this phenomenon; we believe that the online format of the PSRT is not suitable for all professionals. A way to simplify the use of the PSRT could be to integrate it into the medical software used in general practice, which allows some items (surname, first name, date of birth, medical history, daily treatments) to be prefilled. Thus, the GP will only have to report the characteristics of the ADR, the suspected drugs, the date of introduction and cessation and the disease history. However, 163 new GPs made reports during Period 2, which shows a certain enthusiasm for this type of report. Moreover, it is conceivable that many serious ADRs were not reported. It seems that the PSRT does not significantly contribute to an increase in serious ADR reporting. It will be interesting to explore the demographic characteristics of GPs who use or do not use the PSRT to refine this tool and generalize its usage. The distribution of our data during the two time periods corresponded to an interrupted time series. A segmented regression analysis was considered because this approach is often used in this type of series. However, we did not use this methodological approach because of a significant lack of correlation during each of the two periods. The stability of the observed values within each period made it possible to compare them using a quantitative approach.

The establishment of the PSRT allowed an increase in reporting by the GPs at 5 years without compromising the quality of the reports. It will be interesting to deploy this system at a large scale, which will allow us to validate our results.

Our study revealed that ADR underreporting in general practice is highlighted by the small number of GPs involved in PV. The teaching of PV must be included early during the medical education course. GP awareness of reporting to the CRPV in professional education is essential for preventing ADRs and improving the quality of care.

## Data Availability

The datasets used and/or analysed during the current study are available from the corresponding author upon reasonable request.

## Abbreviations

ANSM: Agence nationale de sécurité du médicament
ADR: Adverse drug reaction
ATC: Anatomical Therapeutic Chemical﻿
CRPV: Regional pharmacovigilance centres
DGP: Department of General Practice
DQ: Details query
EMA: European Medicine Agency﻿
GP: General practitioner
Med﻿DRA: Medical Dictionary for Regulatory Activities
PV: Pharmacovigilance
PSRT: Pharmacovigilance simplified reporting tool
URML: Normandie Normandy Regional Union of Liberal Physiciansl
